# Comparative Assessment of Outcomes in Drug Treatment for Smoking Cessation and Role of Genetic Polymorphisms of Human Nicotinic Acetylcholine Receptor Subunits

**DOI:** 10.3389/fgene.2022.812715

**Published:** 2022-02-10

**Authors:** Ahmet Muderrisoglu, Elif Babaoglu, Elif Tugce Korkmaz, Said Kalkisim, Erdem Karabulut, Salih Emri, Melih O. Babaoglu

**Affiliations:** ^1^ Department of Pharmacology, Faculty of Medicine, Hacettepe University, Ankara, Turkey; ^2^ Department of Chest Diseases, Faculty of Medicine, Hacettepe University, Ankara, Turkey; ^3^ Department of Biostatistics, Faculty of Medicine, Hacettepe University, Ankara, Turkey

**Keywords:** smoking, nicotine addiction, smoking cessation, CHRNA, nicotinic acetylcholine receptor alpha subunit

## Abstract

**Objective:** To investigate the effects of genetic polymorphisms of human nicotinic acetylcholine receptor subunits α3, α4 and α5, which are encoded by *CHRNA3*, *CHRNA4 CHRNA5* genes, respectively, on nicotine addiction and outcomes of pharmacological treatments for smoking cessation.

**Methods:** A total of 143 smokers and 130 non-smokers were included. Genotyping for *CHRNA3 rs578776, CHRNA4 rs1044396-rs1044397, CNRNA5 rs16969968* polymorphisms was performed by PCR, flowed by RFLP. Clinical outcomes and success rates of pharmacological treatments for smoking cessation with nicotine replacement therapy (NRT), bupropion or varenicline were determined at the 12th week of the treatment.

**Results:** Overall, 52 out of 143 (36.4%) smokers who received pharmacotherapy were able to quit smoking. Success rates for smoking cessation were similar for female (30.3%) and male (41.6%) subjects (*p* = 0.16). The success rate for smoking cessation treatment with varenicline (58.5%) was significantly higher as compared to other treatments with NRT (20.0%), bupropion (32.3%) or bupropion + NRT (40.0%) (chi-square test, *p* = 0.001). Smoker *vs*. non-smoker status and the clinical outcomes of drugs used for smoking cessation were found similar in subjects carrying wild-type and variant alleles of human nicotinic acetylcholine receptor α subunits.

**Conclusion:** In this study, smoking cessation treatment with varenicline was significantly more effective than treatments with nicotine replacement or bupropion in a cohort of Turkish subjects. Smoker/non-smoker status and the clinical outcomes of treatment with pharmacological agents were similar in subjects with wild-type or variant alleles for human nicotinic acetylcholine receptor subunits α3 (*CHRNA3*), α4 (*CHRNA4*) and α5 (*CHRNA5*).

## Introduction

Nicotine dependence is a very serious health problem and a leading cause of preventable death in many countries. There is an increase in cigarette consumption rates both in the world (WHO, 2021) and in Turkey. Overall, 26.5% of the total population of Turkey were reported as smokers in 2016 (TSI, 2016). In the same year, 58,631 smokers and, 7,941 non-smokers died because of smoking-related causes (Institute of Health Metrics and Evaluation (IHME), 2016).

Nicotine exerts its abusive effects by stimulating neuronal nicotinic acetylcholine receptors (nAChR) and by participating in cholinergic system functions, which regulate emotion, cognition and rewarding effects. nAChRs are ligand-gated ion channels consisting of five subunits that modulate the release of neurotransmitters (Wen et al., 2016). Upregulation of nAChRs, particularly α_4_β_2_ sub-type is important in the development of nicotine addiction (Miwa et al., 2011). Also, α_3_ and α_5_ auxiliary subunits play a role in the modulation of addiction (Fowler et al., 2011).

Fagerström Test for Nicotine Dependence (FTND) has been used for determining the severity of nicotine dependence (Fagerstrom and Schneider, 1989). According to ENSP Guidelines for Treating Tobacco Dependence, FTND scores between 0-3 are classified as low level of dependence, scores between 4-6 as medium and scores 7 or higher as high level of dependence. Pharmacotherapy is available and recommended for high and medium level nicotine-dependent smokers (ENSP, 2020). Among strategies for the treatment of nicotine addiction, pharmacotherapy and psychological counselling are the main ones and pharmacotherapy has been shown to be more effective than other interventions (Kalkhoran et al., 2018). Nicotine replacement therapy (NRT), bupropion (a serotonin-dopamine re-uptake inhibitor) and varenicline (an α_4_β_2_ nAChR partial agonist) are the first-line therapeutics used for smoking cessation. Bupropion and NRT can be used together as a combination treatment (Cahill et al., 2013). Although there has been some advancement in the treatments, clinical success rates remain modest. Absolute smoking cessation rates have been reported to be between 5 and 35% depending on the strategy used (Benowitz, 2009).

It has been reported that success rates of cessation treatments may be altered by pharmacogenetic factors (Lerman et al., 2006; Benowitz, 2008; Bergen et al., 2013; Chenoweth and Tyndale, 2017). Therefore, pharmacogenetic optimization of cessation treatments may potentially improve smoking cessation rates (Chenoweth and Tyndale, 2017). Twin studies indicated that the heritability estimate of smoking cessation is around 50% (Xian et al., 2003; Lessov et al., 2004). Genetic factors, which are indicated in affecting nicotine dependence, include genetic variants of α_3,_ α_4_ and α_5_ subunits of nicotinic receptors, which are encoded by *CHRNA3, CHRNA4* and *CHRNA5* genes, respectively (Saccone et al., 2007; Chenoweth and Tyndale, 2017).

*CHRNA3* rs578776 polymorphism was reported to be associated with change in nAChR functioning (Wang et al., 2009; Wen et al., 2016), and with nicotine dependence levels (Saccone et al., 2009a), while such associations could not be shown by some other studies (Hubacek et al., 2014; Tyndale et al., 2015). A study in the Chinese population indicated that *CHRNA4* rs1044396 and rs1044397 were associated with nicotine dependence, and *CHRNA4* rs1044396 with smoking initiation (Chu et al., 2011). Moreover, a Brazilian study found an association between *CHRNA4* rs1044396 variant and smoking cessation rates in subjects with varenicline therapy (Rocha Santos et al., 2015). Most of these results have not been replicated by other studies (Chenoweth and Tyndale, 2017). It was reported that *CHRNA5 rs16969968* polymorphism causes disruption of α5 nAChR signaling that resulted in the diminishing of stimulatory effects of nicotine (Fowler et al., 2011). Some clinical studies have identified polymorphic A allele of *CHRNA5 rs16969968* as a risk factor for high nicotine dependence in Caucasians (Tobacco and Genetics, 2010; Ware et al., 2011; Chenoweth and Tyndale, 2017). However, other clinical studies found no association between *CHRNA5* rs16969968 genetic polymorphism and success of smoking cessation treatments (Chen et al., 2012; Bergen et al., 2013; Tyndale et al., 2015).

We previously examined the effects of genetic polymorphisms of a few pharmacokinetic targets, namely metabolizing enzymes of CYP2A6, CYP2B6, and the drug transporter ABCB1 (MDR1) on smoking status and success of smoking cessation therapies in a similar but smaller cohort of Turkish subjects (Muderrisoglu et al., 2020). In the current study, within a population of Turkish subjects with an extended number of patients we aimed to investigate the effects of polymorphic variants of a few pharmacodynamics targets; namely human nicotinic acetylcholine receptor subunits α3, α4 and α5 on smoking status and the clinical outcomes of smoking cessation with pharmacotherapies.

## Materials and Methods

### Subjects

This study was reviewed and approved by Hacettepe University Ethics Committee (GO-14/416-03). The patients and participants provided their written informed consent to participate in this study.

Participants were divided into two groups as smokers and non-smokers. Smokers (*n* = 143; 66 females, 77 males) were recruited from subjects who applied to the Smoking Cessation Clinic, Department of Chest Diseases, Hacettepe University for smoking cessation between August 2016 and November 2020. The control group of 130 volunteers (73 females, 57 males) were non-smokers. All participants were aged between 18 and 71. Exclusion criteria for the study were as follows: having a serious heart, liver or kidney disease, using or having an addiction history for products other than nicotine, having a severe anxiety disorder and being pregnant. Fagerström Test for Nicotine Dependence (FTND) was used to determine the severity of nicotine dependence in smokers. Exhaled CO levels were measured by using piCO Smokerlyzer (Bedfont Scientific Ltd., Kent, United Kingdom) to verify smoking status. Subjects with CO values higher than 4 ppm were interpreted as active smokers. Routine drug treatments according to the standard procedures indicated by the ENSP Guidelines for Treating Tobacco Dependence were administered to smokers (ENSP, 2020). Non-smokers were comprised of individuals who never smoked in their lifetime.

Smokers were divided into 4 groups according to the drug treatment and they were prescribed according to the guidelines published by ENSP: NRT (*n* = 40), bupropion (*n* = 47), bupropion + NRT (*n* = 15), and varenicline users (*n* = 41). NRT group was comprised of both patch and gum users. On the 12th week of the drug treatment, smokers were contacted by phone and inquired whether they were able to quit smoking or not.

### Genotyping

Whole Blood DNA Purification Kit (Thermo Fischer Scientific, Waltham, Massachusetts, USA) was used to extract DNA from venous blood. Genotyping was performed by a PCR-RFLP method for the *CHRNA3* rs578776, *CHRNA4* rs1044396- rs1044397 and *CHRNA5* rs16969968 polymorphisms. Previously described methods were modified for genotyping *CHRNA3* rs578776 and *CHRNA5* rs16969968 polymorphisms (Hubacek et al., 2014). New primers were designed for the *CHRNA4* polymorphisms. 200 µM of each dATP, dCTP, dGTP, dTTP, 2.5 mM MgCl_2_, BSA, 12.5 pmol of each primer, 1 unit of Taq DNA Polymerase and 100 ng of genomic DNA with a volume of 25 µl used as PCR mixture (Solis BioDyne, Tartu, Estonia). Hot start PCR conditions were as follows; 95°C for 15 min following 30 cycles of 95°C for 20 s, 60°C for 30 s, 72°C for 1 min, and 72°C for 10 min. A thermal cycler (Bio-Rad T100 Thermal Cycler, Bio-Rad Laboratories, Taipei, Taiwan) was used to perform PCR cycles. PCR products were cut by restriction enzymes (New England Biolabs, Ipswich, Massachusetts, USA). Restriction products were separated by 2–3% agarose gel electrophoresis and UV light was used to visualize fragments (Kodak, Rochester, New York, USA). Details of genotyping methods are provided in Table 1.

**TABLE 1:** Genotyping methods for the *CHRNA3* rs578776, *CHRNA4* rs1044396- rs1044397 and *CHRNA5* rs16969968 polymorphisms.

Gene	Genetic polymorphism	PCR primers	PCR product size	Restriction enzyme	Restriction fragments (bp)	Allele
*CHRNA3*	rs578776	5′-TTC​TTT​ACT​GGG​TCT​AAA​GGG​CTA​TGC​C-3′	146 bp	*NlaIII*	82 + 54+10	C
		5′-ATC​CAC​CCA​GTT​TAT​GGT​GTA​CTA​AG-3′			92 + 54	T
*CHRNA4*	rs1044396	5′-CTT​TGG​TGC​TGC​GGG​TCT​T-3′	84 bp	*HinP1I*	57 + 27	G
		5′-AGC​CCT​CTC​CGT​GCA​AAT​G-3′			84	A
*CHRNA4*	rs1044397	5′-GTC​TGC​AAT​GTA​CTG​GAC​GC-3′	97 bp	*HinP1I*	69 + 28	C
		5′-CAC​GGT​CAA​GAC​CCG​CAG-3′			97	T
*CHRNA5*	rs16969968	5′ -ATG​AAG​AAG​TCA​TGT​AGA​CAG​GTA​CTT​C-3	165 bp	*Tag* ^*α*^ *I*	97 + 68	G
		5′ -TAC​ACA​TCA​CAG​ACC​TCA​CGG​ACA​TC-3			165	A

bp, base pairs.

### Sample Size Calculation and Data Analysis

To predict approximate sample sizes for study groups, we used the Power and Sample Size Program software (Dupont and Plummer, 1990). We applied previously reported frequencies for the variant alleles of interest in Caucasians as 28.1% for the *CHRNA3* rs578776, 52.9% for the *CHRNA4* rs1044396-1044397 and 36.6% for the *CHRNA5* rs16969968 polymorphisms (Genomes Project et al., 2015). The estimated sample sizes were calculated while the type I error probability and the power were set to 0.05 and %80, respectively. With a proposed relative risk of 1.5 for allelic distributions between smokers *vs*. non-smokers and for a study of independent cases and controls with at least one control per case, calculated numbers for cases were 180 subjects for the rs578776 polymorphism, 49 subjects for the rs1044396 and the rs1044397, and 115 subjects for the rs16969968 polymorphisms. Sample sizes needed for each group appeared to be adequate with the estimated numbers for all, but one of the genetic variants examined.

Genotype, haplotype frequencies and gender groups were analyzed by using Chi-Square and Fischer’s exact tests. For multiple comparisons Bonferroni’s correction was applied. For estimation of *CHRNA* haplotype frequencies and their effects on smoking status and cessation rates, SNPStats internet resource was used as an *in silico* statistical tool (SNPStats, 2016). *p* < 0.05 was accepted as a statistical significance level. Statistical analyses were performed by using GraphPad Prism version 6.01 for Windows (GraphPad Software, La Jolla, California, USA).

## Results

### Demographics, Smoking Status and Cessation Rates

Demographic variables are shown in Table 2. There were no statistically significant differences between smokers and non-smokers regarding the genders and ages. Thirty-two (41.6%) of male smokers and 20 (30.3%) of female smokers, and overall 52 (36.4%) smokers were able to quit smoking after 12 weeks from the beginning of the drug treatment.

**TABLE 2:** Demographics and number of individuals in each group according to smoking status.

	Smokers n, (%)	Non-smokers n, (%)	*p*-values
	(Total *n* = 143)	(Total *n* = 130)	
**Gender**
Male	77, (53.8)	57, (43.8)	0.10
Female	66, (46.2)	73, (56.2)	
**Age**	39.2 ± 1.1 (37-41.4)	41.6 ± 1.1 (39.4-43.8)	0.14
**CO (ppm)**	14.2 ± 0.8 (12.7-15.7)	N/A	N/A

Ages are shown as mean ± standard errors of means, S.E.M., and (95% Confidence Intervals).

N/A, not applicable.

**FIGURE 1:**
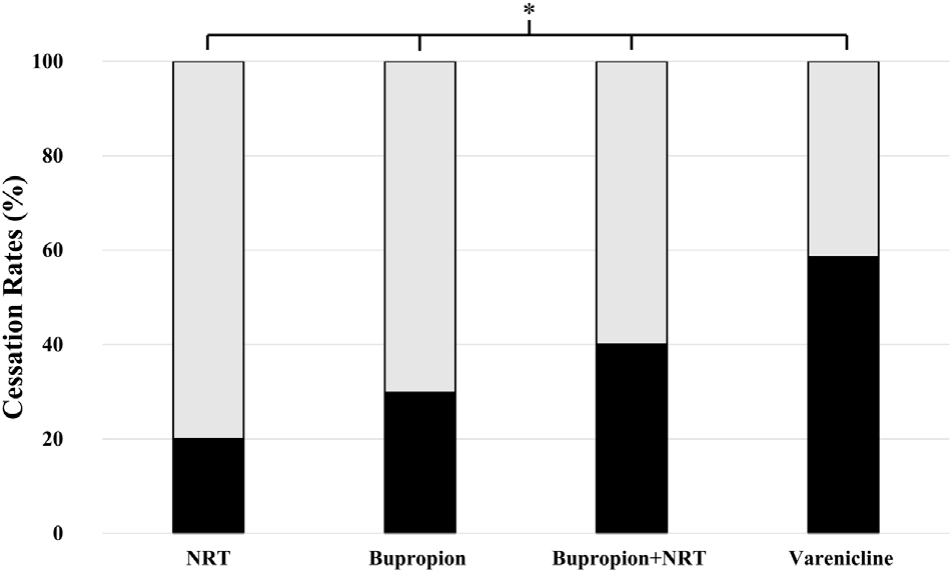
Smoking cessation rates among drug-treatment groups. Cessation rates were as follows: 20.0% (95% CI= 7.6-35.2) for NRT users (*n* = 40), 29.8% (16.7-47.6) for bupropion users (*n* = 47), 40% (15.2-77.9) for bupropion + NRT users (*n* = 15) and 58.5% (43.5-88) for varenicline users (*n* = 41) (Chi-square test, *p* = 0.001; χ2, df = 16.2, 3) (■: Quitters, □: Non-Quitters).

Smoking cessation rates were significantly different among four drug treatment groups. The success rates were 20.0% (CI_95%_ = 7.6-35.2) for NRT, 29.8% (CI_95%_ = 16.7-47.6) for bupropion, 40% (CI_95%_ = 15.2-77.9) for bupropion + NRT and 58.5% (CI_95%_ = 43.5-88) for varenicline users (Figure 1, *p* = 0.001). Post-hoc analysis with Bonferroni’s correction revealed that varenicline treatment was significantly more successful than NRT (*p* = 0.0002) and bupropion treatments (*p* = 0.003).

### Smoking Status and Variants of *CHRNA*

Overall, the frequencies for the examined variant alleles in our study population were 37.2% for the *CHRNA3* rs578776, 48.4% for the *CHRNA4* rs1044396, 46.7% for the *CHRNA4* rs1044397 and 40.8% for the *CHRNA5* rs16969968 polymorphisms. The distribution of all genetic variants examined was consistent with the Hardy–Weinberg equilibrium (*p* > 0.05). The genotype and allele distributions of the polymorphic alleles are summarized in Table 3. As shown, the distribution of the variant alleles and genotypes were similar for all polymorphisms between smokers and non-smokers (Table 3). Therefore, *CHRNA3, CHRNA4* and *CHRNA5* polymorphisms were not associated with smoking status.

**TABLE 3:** Genotype and allele frequencies for the *CHRNA3* rs578776, *CHRNA4* rs1044396, *CHRNA4* rs1044397 and *CHRNA5* rs16969968 polymorphisms in smokers *vs*. non-smokers.

Gene	Genetic polymorphism	Genotypes and alleles	Smokers: n, (%)	Non-smokers: n, (%)	*p*-value (χ2, df)
*CHRNA3*	rs578776	CC	58, (40.6)	51, (39.2)	0.97 (0.0563, 2)
		CT	65, (45.5)	60, (46.2)	
		TT	20, (14)	19, (14.6)	
		C	181, (63.3)	162, (62.3)	0.81 (0.0559, 1)
		T	105, (36.7)	98, (37.7)	
*CHRNA4*	rs1044396	GG	38, (26.6)	46, (35.4)	0.07 (5.33, 2)
		GA	69, (48.3)	45, (34.6)	
		AA	36, (25.2)	39, (30)	
		G	145, (50.7)	137, (52.7)	0.64 (0.217, 1)
		A	141, (49.3)	123, (47.3)	
*CHRNA4*	rs1044397	CC	39, (27.3)	46, (35.4)	0.23 (2.96, 2)
		CT	70, (49)	51, (39.2)	
		TT	34, (23.8)	33, (25.4)	
		C	148, (51.7)	143, (55)	0.45 (0.579, 1)
		T	138, (48.3)	117, (45)	
*CHRNA5*	rs16969968	GG	47, (32.9)	44, (33.8)	0.87 (0.268, 2)
		GA	73, (51)	68, (52.3)	
		AA	23, (16.1)	18, (13.8)	
		G	167, (58.4)	156, (60)	0.70 (0.146, 1)
		A	119, (41.6)	104, (40)	

### Cessation Rates and Variants of *CHRNA*

The distribution of the wild-type and the variant alleles for the *CHRNA3* rs578776, *CHRNA4* rs1044396, *CHRNA4* rs1044397, *CHRNA5* rs16969968 genetic polymorphisms are presented in Table 4. The polymorphic variants were not found to be associated with the success of smoking cessation treatments. Likewise, *CHRNA* haplotypes were not associated with smoking cessation rates (Table 5).

**TABLE 4:** Frequencies of genetic variants between quitters and non-quitters.

Gene	Genetic polymorphism	Allele	Quitters; n, (%)	Non-quitters; n, (%)	*p*-value (χ2, df)
*CHRNA3*	rs578776	C	68, (65.4)	113, (62.1)	0.58 (0.31,1)
		T	36, (34.6)	69, (37.9)	
*CHRNA4*	rs1044396	G	50, (48.1)	95, (52.2)	0.50 (0.45, 1)
		A	54, (51.9)	87, (47.8)	
*CHRNA4*	rs1044397	C	52, (50.0)	96, (52.7)	0.65 (0.2, 1)
		T	52, (50.0)	86, (47.3)	
*CHRNA5*	rs16969968	G	62, (59.6)	105, (57.7)	0.75 (0.101, 1)
		A	42, (40.4)	77, (42.3)	

**TABLE 5:** Estimated *CHRNA* haplotype frequencies among quitters and non-quitters and their association for quitting smoking.

CHRNA3 rs578776	CHRNA4 rs1044396	CHRNA4 rs1044397	CHRNA5 rs16969968	Frequency in quitters	Frequency in non-quitters	Odds ratio (95% CI)	*p*-value
C	A	T	A	0.1869	0.2054	1	—
C	G	C	A	0.1623	0.1884	1.33 (0.43–4.12)	0.62
T	G	C	G	0.1832	0.1827	1.2 (0.47–3.06)	0.71
T	A	T	G	0.0877	0.1616	1.57 (0.42–5.9)	0.51
C	G	C	G	0.1147	0.1419	1.15 (0.41–3.19)	0.79
C	A	T	G	0.1605	0.0853	0.64 (0.21–1.91)	0.42
T	A	T	A	0.0443	0.0203	1.17 (0.06–22.3)	0.92
Rare Haplotypes				0.0604	0.0144	0.19 (0.03–1.19)	0.08

Overall Haplotype Association *p* Value: 0.28.

## Discussion

In this study, we sought to investigate whether smoking status and clinical outcomes of pharmacological treatments with nicotine, bupropion or varenicline for smoking cessation were affected by genetic polymorphisms of several pharmacodynamic targets, namely, human nicotinic acetylcholine receptor subunits α3 (*CHRNA3*), α4 (*CHRNA4*) and α5 (*CHRNA5*) in a sample of subjects in Turkish population. For this aim, a total of 143 smokers and 130 non-smokers were examined.

A routine drug treatment according to the standard procedures as indicated by the ENSP Guidelines for Treating Tobacco Dependence and according to subjects’ FTND scores was administered for each individual (ENSP, 2020). We found out that varenicline treatment was significantly more effective than NRT, bupropion treatment and bupropion + NRT (Figure 1). Historically, the success rate for smoking cessation has been found significantly higher in subjects who were taking any kind of pharmacotherapies, i.e., with nicotine, bupropion or varenicline, as compared to patients with no drug treatment. However, even with pharmacotherapy applications, cessation rates remain modest because of the tenacious nature of nicotine addiction. Frequent relapse of nicotine addiction has also been attributed to the strong character of nicotine dependence (Benowitz, 2009; Kalkhoran et al., 2018). Absolute abstinence should be the goal since it has been reported that even smoking one single cigarette per day increases the risk of cardiovascular diseases and stroke (Bonnie RJ and Kwan, 2015; Hackshaw et al., 2018).

Our findings for drug efficacy were in agreement with previous studies, as bupropion and varenicline were found more effective than NRT alone (Stead et al., 2012; Cahill et al., 2013). Particularly, the findings in the EAGLES trial, which reported that a 6-months abstinence rate was highest among varenicline users, support our finding of superior efficacy with varenicline (Anthenelli et al., 2016). A meta-analysis study reported that varenicline treatment has been shown to improve the chance of quitting more than other therapies (Cahill et al., 2013). Our findings support the view that bupropion or varenicline treatments should be preferred over treatment with NRT-alone in smoking cessation.

Few previous studies reported associations between genetically polymorphic variants of pharmacokinetic and pharmacodynamic targets. Such studies imply that pharmacogenomic data may facilitate the optimization of drug treatments for smoking cessation (Chenoweth and Tyndale, 2017). We previously studied the effects of genetic polymorphisms of a few pharmacokinetic targets, mainly metabolizing enzymes of CYP2A6, CYP2B6, and the drug transporter ABCB1 (MDR1) on smoking status and success of smoking cessation therapies. In that study, we reported that genetic variants of the drug transporter ABCB1 and a particular haplotype (*1236TT-2677TT-3435TT*) were significantly associated with non-smoking status, while no other associations with genetic variants of ABCB1 or CYP2A6, CYP2B6 with nicotine addiction was found (Muderrisoglu et al., 2020). Therefore, in the current study we aimed to investigate whether the smoking status or the clinical outcomes of pharmacological treatments for smoking cessation might be affected by genetic polymorphisms of pharmacodynamic targets of human nicotinic acetylcholine receptor subunits α3 (*CHRNA3*), α4 (*CHRNA4*) and α5 (*CHRNA5*) subunits.

The SNPs examined were selected on the basis of their functional effects on receptor expression and functioning (as explained in Table 6) and their frequent occurrence among Caucasians. In previous studies, frequencies for the variant alleles in Caucasians were found to be 28.1% for the *CHRNA3* rs578776, 52.9% for the *CHRNA4* rs1044396, 52.9% for the *CHRNA4* rs1044397 and 36.6% for the *CHRNA5* rs16969968 polymorphisms (Genomes Project et al., 2015). We did not find any association of *CHRNA* polymorphisms with either smoking status or clinical success of pharmacotherapies for smoking cessation (Tables 3, 4 and 5).

**TABLE 6:** Summary of the polymorphic variants examined and their association with previously reported functional consequences.

Gene	SNP	Location	Variant Type	Functional Consequences
*CHRNA3*	rs578776	chr15:78596058, intron 1 of *CHRNA3*	3′ Prime UTR Variant	No amino acid substitution; a tag SNP associated with the change in mRNA expression of *CHRNA5* (Wang et al., 2009; Wen et al., 2016)
*CHRNA4*	rs1044396	chr20:63349782, exon 5 of *CHRNA4*	Missense Variant	Amino acid substitution from serine to arginine at position 543 of the α4 subunit of nAChR; alteration of α4β2 nAChR sensitivity (Li et al., 2005; Winterer, 2011)
*CHRNA4*	rs1044397	chr20:63349752, exon 5 of *CHRNA4*	Synonymous Variant	No amino acid substitution; change of electrophysiological properties of the nicotinic receptor subtype α4β2 (Winterer, 2011; Mobascher et al., 2016)
*CHRNA5*	rs16969968	chr15:78590583, exon 5 of *CHRNA5*	Missense Variant	Amino acid substitution from aspartate to asparagine at position 398 of the α5 subunit of nAChR; a greater influx of calcium ions and disruption of α5 nAChR signaling (Fowler et al., 2011)

*CHRNA3* is responsible for coding the α3 auxiliary subunit of nAChRs. This subunit regulates the function of nAChRs (Miwa et al., 2011). rs578776 is located 3′-UTR region of the *CHRNA3* gene and found to be associated with mRNA expression of *CHRNA5* (Wen et al., 2016). Some of the previous similar studies reported associations between *CHRNA3* rs578776 polymorphism and nicotine dependence in Caucasians (Saccone et al., 2009b; Tyndale et al., 2015; Hubacek et al., 2017). These studies also reported that the treatment outcomes for smoking cessation were not affected by *CHRNA3* rs578776 polymorphism. In contrast, a Chinese study reported the C allele of *CHRNA3* rs578776 polymorphism was significantly related to smoking cessation (Wang et al., 2016). Another study by Chaity et al. (2021) reported that *CHRNA3* rs578776 polymorphism was associated with smoking status in a Bengali population (Chaity et al., 2021). However, unlike the Caucasians, no association between *CHRNA3* rs578776 polymorphism and nicotine dependence was detected in Africans (Saccone et al., 2009b). Our results of the current study in a Caucasian Turkish population yielded a lack of association between *CHRNA3* rs578776 polymorphism and smoking status or success of smoking cessation.

It was reported that the majority of the high-affinity nicotine-binding sites were present on the α_4_β_2_ sub-type of nAChRs in the brain (Flores et al., 1992; Lindstrom, 1997). A previous study found that activation of the α_4_ subunit of nAChRs is sufficient for the development of reward, tolerance and sensitization mediated by nicotine (Tapper et al., 2004). It has been suggested that genetic polymorphisms of *CHRNA4* exert functional consequences by altering receptor function or mRNA expression (Li et al., 2005). rs1044396 causes an amino acid substitution from serine to arginine at position 543 of the α4 subunit of nAChR (Li et al., 2005). rs1044397 is a synonymous variant and causes no amino acid substitution; however, it has been linked with a change of electrophysiological properties of the nicotinic receptor subtype α4β2 (Mobascher et al., 2016). A previous study measured electrophysiological properties of the nicotinic receptor subtype α_4_β_2_ in animals carrying the combined haplotype for both of the C*HRNA4* rs1044396 and rs1044397 variants and reported that this haplotype altered α_4_β_2_ nAChR sensitivity (Winterer et al., 2011). An association between the variant (A) allele of *CHRNA4* rs1044396 and psychiatric illnesses such as depression, anxiety disorders and certain other addictions was reported (Markett et al., 2011; Tsai et al., 2012; Jeong et al., 2017). As widely known, most smokers report alleviation of their anxiety with smoking (Brandt, 2007) and nicotine has cognitive enhancer activity (Davis and Gould, 2009). Therefore, it may be conceivable that *CHRNA4* polymorphisms may alter smoking behavior *via* altering cholinergic system functions related to mood change and cognition. Recently, Gu et al. (2020) found that homozygous AA genotype carriers of *CHRNA4* rs1044396 polymorphism were more likely to quit smoking after the diagnosis of lung cancer in their cohort of Chinese patients (Gu et al., 2020). Also, there are studies in the literature that suggested associations of *CHRNA4* rs1044396 with outcomes of bupropion and varenicline treatments (Feng et al., 2004; King et al., 2012; Rocha Santos et al., 2015). In contrast, the associations could not be found by some other studies (Chenoweth and Tyndale, 2017). Our results confirm the lack of association between *CHRNA4* rs1044396 or *CHRNA4* rs1044397 polymorphisms with smoking status or quitting rates in cessation interventions in Caucasians.

*CHRNA5* rs16969968 polymorphism causes amino acid substitution from aspartate to asparagine at position 398 of the α5 subunit of nAChR protein sequence and a previous study on knockout mice reported that the amino acid change resulted in a greater influx of calcium ions and disruption of α5 nAChR signaling (Fowler et al., 2011). Their findings suggest that as a result of α5 nAChR signaling disruption, stimulatory effects of nicotine diminishes on the medial habenulo-interpeduncular nucleus pathway. Thereby, greater quantities of nicotine are needed to gain stimulatory effects. (Fowler et al., 2011). In line with this finding, clinical studies identified polymorphic A allele of *CHRNA5* rs16969968 as a risk factor for higher nicotine dependence in Caucasians (Tobacco and Genetics, 2010; Ware et al., 2011; Chenoweth and Tyndale, 2017). Moreover, a meta-analysis study by Chen et al. (2015) examined associations between rs16969968 and age of quitting smoking, age of lung cancer diagnosis in 24 studies of European ancestry. They reported that the rs16969968 variant allele (A) was associated with a lower likelihood of smoking cessation and the AA genotype was associated with a 4-year delay in the median age of quitting compared with the GG genotype, and homozygous variant AA genotype carriers for the *CHRNA5* rs16969968 polymorphism had a 4-years earlier age of diagnosis of lung cancer due to smoking (Chen et al., 2015). A recent study by Hopkins et al. (2021) demonstrated that *CHRNA5* rs16969968 AA genotype independently associated with smoking exposure and its main complications i.e., chronic obstructive pulmonary disease and lung cancer (Hopkins et al., 2021). In contrast, in African-Americans, an association between *CHRNA5* rs16969968 polymorphism and smoking behavior was not detected (Zhu et al., 2014). Our results in a different Caucasian population of Turkish subjects yielded no association between *CHRNA5* rs16969968 and smoking or cessation status. This discrepancy may be explained by relatively small sizes of samples in the studies, as well as differences in ethnicities. Indeed, Tyndale et al. (2015) and Chenoweth and Tyndale (2017), suggested that *CHRNA5* rs16969968 polymorphism did not affect the success of pharmacotherapies with smoking cessation (Tyndale et al., 2015; Chenoweth and Tyndale, 2017). Also, Breitling et al. (2009) indicated that neither *CHRNA3* rs578776 nor *CHRNA5* rs16969968 was associated with smoking cessation (Breitling et al., 2009). Also, a recent study by Hubacek et al. (2021) demonstrated no association of polymorphisms of *CHRNA3* rs578776 and *CHRNA5* rs16969968 between nicotine dependence, treatment success and nicotine metabolite concentrations (Hubacek et al., 2021). Their findings in Caucasian populations are in agreement with our results in our cohort of Turkish population that suggested lack of association of *CHRNA5* rs16969968 with smoking cessation treatments outcomes.

A limitation of our study is that our study was conducted in a relatively small size of population sample with 143 smokers and 130 non-smoker control subjects. While our finding of superior clinical efficacy of varenicline treatment in smoking cessation has consistently been reported also by other studies in literature (see Cahill et al., 2013; Anthenelli et al., 2016), larger sample sizes in further studies would probably yield clearer results for comparison of effectiveness of drug molecules used for this purpose. Also, studies with larger sample sizes may provide more conclusive results for addressing influence of *CHRNA* variants on smoking status and success of cessation. Another limitation is that we could follow up smokers only by phone-calls at the 12th week, but not afterwards and with a way of communication that could have provided a face-to-face interaction with subjects. It is well-known that smoking cessation rates would decrease over time.

In conclusion, in the present study we report that varenicline treatment was significantly more effective when compared to bupropion or nicotine replacement treatments in smoking cessation. Our results yielded informative, yet inconclusive results for association between genetic polymorphisms of α3, α4 and α5 subunits of nicotinic acetylcholine receptors with either smoking status or clinical outcomes of pharmacotherapies for smoking cessation. Nevertheless, we believe that the present study may be helpful in understanding the nature of smoking habit and the association of smoking cessation with pharmacogenomics of nicotinic acetylcholine receptor subunits.

## Data Availability

The data presented in the study are deposited in the Mendeley data repository, accession number: https://doi.org/10.17632/pjf5rdcdf9.1.
